# Local Heat Stroke Prevention Plans in Japan: Characteristics and Elements for Public Health Adaptation to Climate Change

**DOI:** 10.3390/ijerph8124563

**Published:** 2011-12-07

**Authors:** Gerardo Sanchez Martinez, Chisato Imai, Kanako Masumo

**Affiliations:** 1 Basque Centre for Climate Change, 4 Alameda Urquijo, Bilbao Vizcaya 48008, Spain; 2 World Health Organization Centre for Health and Development, 1-5-1 Wakinohama-Kaigandori Chuoku, Kobe 651-0073, Japan; Email: imaic@wkc.who.int; 3 Graduate School of Medicine, University of Tokyo, 7-3-1 Hongo, Bunkyoku 113-0033, Tokyo, Japan; Email: masunok@m.u-tokyo.ac.jp

**Keywords:** local governments, heat-health action plans, climate change, adaptation

## Abstract

The adverse health effects from hot weather and heat waves represent significant public health risks in vulnerable areas worldwide. Rising temperatures due to climate change are aggravating these risks in a context of fast urbanization, population growth and societal ageing. However, environmental heat-related health effects are largely preventable through adequate preparedness and responses. Public health adaptation to climate change will often require the implementation of heat wave warning systems and targeted preventive activities at different levels. While several national governments have established such systems at the country level, municipalities do not generally play a major role in the prevention of heat disorders. This paper analyzes selected examples of locally operated heat-health prevention plans in Japan. The analysis of these plans highlights their strengths, but also the need of local institutions for assistance to make the transition towards an effective public health management of high temperatures and heat waves. It can also provide useful elements for municipal governments in vulnerable areas, both in planning their climate change and health adaptation activities or to better protect their communities against current health effects from heat.

## 1. Introduction

Climate change is already affecting health, and the nature and extent of such effects are becoming increasingly clear [1]. Despite the necessary mitigation efforts through the curbing of greenhouse gas (GHG) emissions, some health-relevant impacts of climate change are thought to be inevitable at this point [2]. Thus, communities and institutions around the world will have to adapt to those impacts. Subnational governments and especially municipalities are closer to where the impacts of climate change will be most acutely felt. Therefore, they are in a key position to design and implement policies to adapt to climate change, and in particular to protect the local populations from its associated health risks. 

Many local governments have been highly proactive in tackling climate change and preventing its impacts, even in countries where the national government has been reluctant to take significant steps in that direction [3]. However, the involvement of local authorities in climate change policies is still scarce in general. A number of obstacles make such involvement difficult, both for GHG mitigation and adaptation activities [4]. Firstly, local governments are not generally subjected to direct international pressure or binding agreements regarding climate change, although several have enrolled voluntarily in transnational municipal networks [5]. They often do not have the authority or competencies to engage in certain types of climate change-relevant policies. Smaller or resource-constrained municipalities may lack the financial means and capacity to design or implement several types of actions, for instance adaptation based on infrastructure (e.g., Dams to protect against sea level rise). Lastly, in a context of competing priorities, climate change might be low on the local political agenda, and municipalities would rather let other levels of governance deal with the issue. 

Despite such obstacles, there are several opportunities for local governments to effectively engage in climate change policies, in particular for adaptation. Specifically, public health adaptation to climate change impacts might represent “low hanging fruit” for municipalities willing to engage in efforts towards climate change adaptation in general. 

Prevention against heat waves is a good example. Heat-related mortality and diseases have increased in the last decades and are expected to worsen with climate change [6]. Long recognized as a threat to human health, recent extremely deadly heat episodes (such as the summer 2003 heat waves in Western Europe) brought the issue to the center of public attention [7,8,9]. They also brought attention to the general lack of preparedness for such impacts [10,11]. Moreover, climate change is making heat wave episodes more frequent, longer and more intense in general, and heat-related mortality and morbidity is projected to increase under most considered long-term scenarios [6,12].

Generally after heat waves resulting in high mortality, governments around the world have taken preventive measures. Heat Wave Warning Systems (HWWS), combined with public health prevention and education, have proved effective in averting a significant proportion of heat-related deaths and disease [13,14]. Heat-health prevention is most effective when implemented at various government levels, from national to local. However, local involvement in public health prevention of heat waves is still not widespread [15]. We corroborated this imbalance while conducting research for a subnational government in Japan that sought to strengthen its public health prevention against heat waves.

Albeit not exceptional, the case of Japan illustrates both the worsening heat waves and health trend and the lack of local involvement in its prevention. Geographical and demographic particularities further compound the vulnerability of the country and its cities. While the Japanese archipelago encompasses various climatic zones, most of its population—consisting of about 127 million—lives in temperate areas prone to periodic hot spells. The population concentrates in a small proportion—about 3% of the land, mostly cities [16,17].

In line with international experience most heat-related deaths in Japan usually happen in large cities (notably Tokyo, Osaka and Nagoya), though smaller urban areas have also had significant heat mortality in the last decades. Indeed, typical urban settings in Japan combine factors known to increase the health effects of heat waves, including a marked “urban heat island” effect and a rapidly aging population, adding to the pool of individuals at higher risk [18,19]. From 1959 to 2003 the mortality rate from heat disorders was 0.10 per 100,000 population a year on average [20]. However, this heat-related mortality has increased dramatically in the last decades. Recent peaks of heat-related mortality in major Japanese cities were registered in the years 1978, 1983, 1990 and 1994 [21]. The average yearly mortality rate from heat stroke from 1999 to 2003 was 0.23 per 100,000 population [22]. 

Official statistics suggest a worsening situation. Ambulance records since 2003 show a steady increase in the number of heatstroke patients in the 17 largest cities in the country [23,24]. Further, climate change is likely to increase public health risks from heat waves in Japan in coming years. A computerized global warming simulation determined that the yearly number of days over 30 °C will grow almost three-fold on average for the whole country by the year 2100, even if strong action at the international level is taken to curb climate change [25]. 

Confronted with this situation, national authorities in Japan have implemented several initiatives and programs to prevent the health effects from high temperatures. The Ministry of the Environment has developed and distributes guidelines to prevent heat-related diseases, with specific recommendations for prevention at the local and regional levels [26]. The Ministry of Health, Labor and Welfare (MOHLW) also promotes preventive action on heat through health education activities and the distribution of health promotion materials. These and other national entities maintain websites on the prevention of heat disorders. Forecasts of Wet Bulb Globe Temperature (WBGT—a widely used heat stress index) and levels of heat disorder risk are provided by the National Institute of Environmental Studies (NIES) throughout the summer via the Internet. 

These national activities provide ample theoretical and applied basis for the adoption of preventive action by prefectures and municipalities. However, Heat-health action plans or programs managed or participated in by local governments are still few, particularly in smaller cities where lack of know-how and resource constraints might steer preventive action towards other areas. 

Despite these challenges, some municipalities in Japan have undertaken plans or programs to prevent heat disorders at the local levels. Other municipalities in Japan and abroad can learn from their experiences to develop their own preventive programs and public health responses to heat waves. The present paper aims at finding useful lessons through the comparative analysis of publicly available information on selected locally operated heat disorder prevention plans in Japanese cities. Furthermore, the adoption of operated programs for prevention of health effects during heat waves (where relevant) can be an entry point or first step for municipalities wishing to increase their involvement in climate change adaptation activities in general. 

## 2. Experimental Section

We collected information on locally run heat-health actions in Japan through an Internet search with a publicly available, free search engine. The search was restricted to Japanese websites, and the following terms (in Japanese) were used: “heat stroke”, “heat waves”, “tropical hot days”, “early warning system”, “heat wave warning”, “high temperatures alert”, “municipality”, “local plan”, “heat alert”, “local government”, “public health prevention” and “heat wave prevention plan”. For comparability of bioclimatic and other factors, the search was limited to plans from cities on the Pacific side of central Honshu Island [27] with a population between 50,000 and 500,000 inhabitants. Because the objective was to find heat-health action plans run by relatively small municipalities, all plans run by cities with more than 500,000 people were not taken into account. 

Further selection criteria were that the plans were: (1) aimed at preventing health effects from environmental (*i.e.*, non-occupational) heat exposure and (2) coordinated and operated by local authorities. We collected additional information through phone interviews with key informants (*i.e.*, responsible officers or personnel directly involved) at the city governments of the analyzed plans. We attempted to expand our search to plans that met the criteria but had not published any information online. However, we could not perform such expanded search due to limitations in time and resources (further details are included in the discussion section).

The information found online was categorized along the elements proposed by the World Health Organization (WHO) [13] as core for a heat-health action plan. These elements are:

1. Agreement on a lead body (to coordinate a multipurpose collaborative mechanism between bodies and institutions and to direct the response if an emergency occurs).
2. Accurate and timely alert systems (heat-health warning systems trigger warnings, determine the threshold for action and communicate the risks).
3. A heat-related health information plan (about what is communicated, to whom and when).
4. A reduction in indoor heat exposure (medium- and short-term strategies) (advice on how to keep indoor temperatures low during heat episodes).
5. Particular care for vulnerable population groups.
6. Preparedness of the health and social care system (staff training and planning, appropriate health care and the physical environment).
7. Long-term urban planning (to address building design and energy and transport policies that will ultimately reduce heat exposure).
8. Real-time surveillance and evaluation.

For a more precise description of the plans, epidemiologic surveillance and evaluation of the plan have been divided into two distinct categories. “Long term urban planning” has been included in the case study, even if related actions pertain to other ongoing plans or programs unrelated to heat stroke prevention. 

We collected additional information on the selected plans through phone interviews with key informants (*i.e.*, responsible officers or personnel directly involved) at the city governments. All responsible officers for the selected plans responded to our questions (see list below). The interviews were used to expand the information found online for each of the elements proposed by WHO. The translation of the structured list of questions asked during the interviews is in Annex 1 (Once the information was categorized, all key informants were re-contacted for confirmation, fact-checking and possible updates).

## 3. Results and Discussion

### 3.1. Results

**Figure 1:**
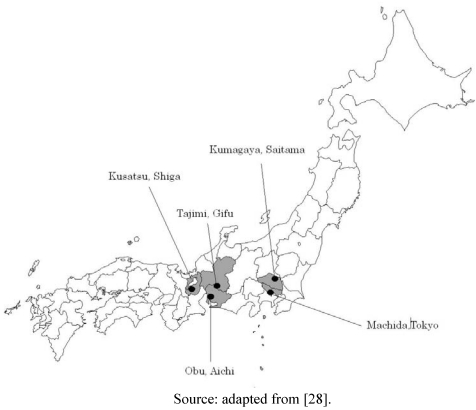
Selected municipalities in Japan that run heat stroke prevention plans.

Five heat-health action plans met the criteria set forth in the experimental section of this article, from the cities of Kusatsu (Shiga), Kumagaya (Saitama), Tajimi (Gifu), Obu (Aichi) and Machida (Tokyo), all located in Honshu (Figure 1). 

The heat disorder prevention activities of these local governments were analyzed to showcase the most relevant characteristics of locally run heat-health preventive systems in small Japanese urban settings. What follows is a summary of the basic characteristics of each plan: 

Kusatsu (Shiga prefecture, approximate 120,000 inhabitants): the municipality runs a “Heat stroke prevention plan” coordinated by the Crisis Management Division. Its heat warning system is based on the automated, real-time monitoring of Wet Bulb Globe Temperature (WBGT) and Air temperature (Celsius) at a local elementary school. Alerts are triggered manually by a local technician when WBGT exceeds 28 °C or air temperature exceeds 31 °C, activating a warning system that cascades information to a comprehensive list of registered citizens and institutions, businesses, media outlets and other relevant stakeholders. The plan encompasses health education activities and specific provisions for outreach to vulnerable groups through volunteer networks. Such networks (“Minsei committees”) are officially recognized and periodically evaluated by prefectural governments. Advice on how to keep cool indoors is distributed through various channels included in the information plan. Long term urban planning for heat island reduction is addressed through measures within a local plan on global warming. The government of the city does not engage in formal epidemiologic surveillance activities, although it does collect data on morbidity and mortality on a yearly basis. The effectiveness of the heat stroke prevention plan is monitored through the use of these outcomes, as well as process indicators which are used to evaluate the plan and reported yearly to selected national institutions and to the general public. 

Kumagaya (Saitama prefecture, approximate 100,000 inhabitants): the city’s “Heat stroke prevention project” is coordinated by the local Department of Health Promotion, based on a heat warning system fed with data from WBGT monitoring systems at elementary schools and the city hall. The Japan Weather Association (JWA) assesses heatstroke risks for the municipality in the following 1 to 21 hours, and reports them categorized by WBGT risk ranks (From 1—Safe to 5—Danger) to a designated focal point in the local government. Different risk level assessments trigger appropriate sections of an information plan directed towards institutions and registered private citizens through various media. Cooling (misting) public places are provided by the authorities when deemed necessary (based on ground air temperature and humidity). There is an active outreach and measures directed towards the care of the elderly living alone through volunteer organizations. There is also a long-term strategy of heat-reducing urban planning and landscape improvement made explicit within the plan. The plan does not include formal epidemiologic data collection activities, although data on the annual number of heatstroke emergency room (ER) confirmed admissions are collected by the local government to evaluate the plan’s effectiveness. 

Tajimi (Gifu prefecture, approximate 117,000 inhabitants): the city’s Health and Welfare division coordinates the “Prevent heat stroke” plan. Warnings are based on an automated WBGT monitoring system placed at an elementary school in the city. A heat risk alert is triggered if WBGT exceeds 28 °C and ambient temperature (as reported by the Japan Meteorological Agency network) exceeds 31 °C. These thresholds were reportedly copied from those in use by other local governments. Upon alert triggering, the warning system automatically cascades information to registered citizens and institutions, businesses and media outlets. There is an active outreach and measures directed towards the care of the elderly living alone through local volunteer organizations. The plan does not include specific provisions for the social and healthcare providers´ preparedness. Long term urban planning for heat island reduction is addressed in the local urban management plans, and heat stroke plan includes a green planting project involving senior citizens. The government of the city does not collect data on heat-related disorders. Informal evaluation of the plan in terms of process is based on the number of participants registered to receive information on heat alerts, reported yearly. 

Obu (Aichi prefecture, approximate 85,000 inhabitants): the heat-health prevention plan is led by the Health and Welfare division, and it is based on an automated WBGT monitoring system maintained and operated by a local university (Shigakukan). The system issues warnings of increasing risk levels coded by colors (Blue, Green, Yellow, Orange and Red) which are distributed through email (including cell phones) only to registered participants including institutions and private citizens. There are some provisions for outreach to vulnerable populations, specifically the elderly through local volunteer organizations. Senior citizens are further involved in the urban management aspects of the plan through a green planting (“green curtain”) project. No other specific provisions are made in the plan for reduction of indoor heat exposure, social and healthcare preparedness, epidemiologic surveillance or monitoring and evaluation. The city government promotes the reduction of GHG emissions through the use of renewable energies, but does not take explicit action on urban management to reduce heat exposure. 

Machida (Tokyo prefecture, approximate 400,000 inhabitants): about 35 km southwest of central Tokyo. The city’s “School Heat Stroke Prevention” program is managed by the local Education Committee, and does not rely on a centralized warning system. Rather, each elementary and middle school in the city is provided with Portable WBGT measurement devices and guidelines on heat stroke risk. The guidelines provide risk ranks along the categories set forth by the Japan Sports Association [29] and instruct teachers to take measures accordingly. All middle schools are provided with fans and portable refrigerators for the provision of ice. In addition, training sessions are provided at the beginning of the summer for teachers in local schools. The plan, focused specifically on school sports, does not address vulnerable groups, social and health care preparedness, long term measures or surveillance. No information could be found on the monitoring and evaluation of the plan. 

This information (summarized in Table 1) is presented along the dimensions proposed by WHO as core elements for a heat-health action plan [13]. 

**Table 1:** Key elements of locally operated heat stroke prevention plans in Japan.

Key element	KUSATSU (Shiga)	KUMAGAYA (Saitama)	TAJIMI (Gifu)	OBU (Aichi)	MACHIDA (Tokyo)
Lead Body	- Crisis Management Division	- Department of Health Promotion	- Crisis Management Division (in collaboration with the local Health Center)	- Division of Health and Welfare (in collaboration with a local University)	- Education committee
Alert System	- Automated WBGT monitoring system at Elementary School (central location in the city), warning manually triggered by local government technician.	- Automated WBGT monitoring systems at elementary schools and the city hall, warning manually triggered by Japan Weather Association according to Japan Sports Association guidelines.	- Automated WBGT monitoring system at a nursery school in the city, warning triggered automatically.	- Automated WBGT monitoring system placed at a local University, warning triggered automatically according to Japan Sports Association guidelines.	- Portable WBGT measurement devices are distributed to elementary and middle schools in the city, warning triggered automatically.
Heat related health information plan	Alert days - Behavioral advice issued according to risk level - Email service (cell phones and PC) for registered addressees - Warning in City government website - Fax to registered organizations, institutes, work places - Advisories in local radio broadcast - Signs and announcements at City Hall - Radio communication system to district councils - Announcements from public vehicles	Alert days - Behavioral advice issued according to risk level - Email service (cell phones and PC) for registered addressees - Warning in City government website - Fax to registered organizations, institutes, work places - Advisories in local TV stations - Signs and announcements at City Hall - When rank 4 warning is triggered, city government calls major senior centres	Alert days - Behavioral advice issued according to risk level - Email service (cell phones and PC) for registered addressees - Radio communication system to district councils - Advisories in local radio broadcast	Alert days - Behavioral advice issued according to risk level - Email service (cell phones and PC) for registered addressees - Warning in City government website	Alert days N/A (Educators trained to avoid outdoors physical activities if WBGT exceeds 31ºC)
	Routinely - Distribution of leaflets - Distribution of prevention guidelines - Heat Stroke Seminars	Routinely - Distribution of leaflets - Banners at public facilities - Public education campaigns	Routinely - Advisories in local newspapers - Public announcement devices - Lectures at Infant clinics - Signs at public facilities, institutions, businesses and offices	Routinely N/A	Routinely - Education manuals annually updated and distributed to sports instructors and school teachers. - Training sessions led by experts in June and July (twice a year).
Reduction in indoor heat exposure	- Green curtain project (cultivation of climbing plants on façades)	- Green curtain project (cultivation of climbing plants on façades)	- Green Curtain Project (cultivation of climbing plants on façades)	- Green Curtain Project for schools and city halls (cultivation of climbing plants on façades) - Fans in elementary and middle schools	- Each middle school is provided with fans and portable refrigerator for ice in gyms.
Care for vulnerable people	- Targeted distribution of informative leaflets - Senior resident halls and clubs are invited to register in heat alert email service.	- Active outreach to the elderly who live alone by social workers - Distribution of leaflets, portable heat measurement devices and special “cooling” scarves - Minsei (civil volunteers) visit seniors on regular basis during hot spells	- Distribution of leaflets to vulnerable population - Minsei (civil volunteers) visit seniors on regular basis during hot spells - Newspaper deliverers inform the city if they find pile of accumulated newspapers in mail box	- City runs a general local support system for senior residents, including heatstroke prevention. - Free 24/7 teleassistance communication devices. - Minsei (civil volunteers) visit seniors on regular basis during hot spells - Newspaper deliverers inform the city if they find pile of accumulated newspapers in mail box	- N/A
Preparedness of the health and social care system	- No specific provisions for health care facilities and/or social services.	- No specific provisions for health care facilities and/or social services	- Health care facilities are periodically recommended to register in heat alert mail service by the city	- Health care and nursing facilities are invited to prepare for hot spells and register in heat alert mail service.	- N/A
Long-term urban planning and GHG emissions reduction	- Global Cooling Project (independent from heat stroke prevention plan), including: 1) Energy conservation plans 2) Ecological Vehicle Promotions 3) Use of solar energy systems 4) Climate campaigning and education.	- City-funded application of reflective painting on buildings and roads - Green roofing (office buildings) - Financial support for tree planting around private and public buildings and houses - Increase in Green public spaces	- Financial support for tree planting around private and public buildings and houses - Installation of reflective materials for roads and housing - Increase in Green public spaces andpublic water bodies and fountains	- Financial support for solar light systems and hybrid water boiling machines (independent from heat stroke prevention plan).	**- N/A**
Surveillance, monitoring and evaluation (M&E)	-Surveillance System: N/A.	-Surveillance System: N/A.	-Surveillance System: N/A.	-Surveillance System: N/A.	-Surveillance System: N/A.
	-M&E: outcome and process indicators collected and reported yearly to national entities, including 1) Number of deaths 2) Ambulance calls for heatstroke 3) Number of mail service registrants and 4) Number of alert days	-M&E: outcome indicator collected and published yearly (ER visits with diagnosis of heatstroke)	-M&E: outcome and process indicators collected and published yearly, including 1) Number of mail service registrants and 2) Heatstroke emergency ambulance transports	-M&E: N/A (currently being planned).	-M&E: N/A

Regarding the specific risk indicators and thresholds used in the plans, all analyzed prevention plans used Wet Bulb Globe Temperature (WBGT) as the indicator for heat risk, along with ambient temperature. The Wet Bulb Globe Temperature (WBGT) is a widely used index for the assessment of heat stress. Primarily developed for industrial settings, it combines three types of measurements [30]:

- Natural wet-bulb temperature (NWBT) measured with a natural wet-bulb thermometer.
- Globe temperature (GT) measured with a black globe thermometer.
- Air temperature (AT) measured with a dry-bulb (normal) thermometer in a shaded area.

For indoor and outdoor conditions with no solar load, WBGT is calculated as: 

                                                WBGT = 0.7NWBT + 0.3GT

For outdoors with a solar load, WBGT is calculated as:

                                         WBGT = 0.7NWBT + 0.2GT + 0.1AT

Although WBGT is mainly used to measure occupational exposures, it has also been used by the military and athletes. In the case of Japan, its use for the general public derives from the early involvement and central role of sports-related associations and sports medicine experts in researching and preventing the effects from heat stress. The thresholds adopted by the local government featured in this article (Table 2) were either taken directly (Kumagaya and Obu) or adapted (Kusatsu, Tajimi and Machida) from the national-level guidelines originally developed by the Japan Sports Association [30].

**Table 2:** Heat alert warning triggering criteria in selected Japanese cities.

City	Warning triggering criteria
Kusatsu (Shiga)	WBGT exceeds 28 °C AND (atmospheric) temperature exceeds 31 °C.
Kumagaya (Saitama)	Rank 1: Safe (<21 °C)
	Rank 2: Attention (≥21 °C)
	Rank 3: Warning (≥25 °C)
	Rank 4: Strong Warning (≥28 °C)
	Rank 5: Danger (≥31 °C)
Tajimi (Gifu)	WBGT exceeds 28 °C AND (atmospheric) temperature exceeds 31 °C.
Obu (Aichi)	Red WBGT(≥31 °C)
	Orange 28 °C≤ WBGT <31 °C
	Yellow 25 °C ≤ WBGT < 28 °C (Warning threshold)
	Green 21 °C ≤ WBGT < 25 °C
	Blue WBGT < 21 °C
Machida (Tokyo)	WBGT exceeds 28 °C AND (atmospheric) temperature exceeds 31 °C.

### 3.2. Discussion

Based on the information publicly available on the Internet and the data provided by key informants at the local governments, some clear characteristics can be derived from the analysis of the five prevention plans featured:

1. There is a clearly identified lead body in charge, or in a coordination role. This body is often, but not always, a local health agency.
2. Real-time monitoring and automated warning systems are preferred over schemes based in medium term forecasting and expert judgment.
3. Wet Bulb Globe Temperature (WBGT) is the preferred indicator for heat risk.
4. The thresholds for the triggering of warnings are either taken directly or adapted from sets of guidelines developed by national institutions.
5. The heat-health information provisions constitute the most developed dimension of the plan, with a wide range of communication strategies and channels. The use of information and communications technologies, particularly email and cell phone notifications to registered participants is a major component within most information plans.
6. Registration of private citizens in the warning distribution lists is voluntary, rather than based on cross-referenced censuses from health centers and/or social services.
7. Health education activities besides alert notifications, leaflets and instructions are a common strategy, but not consistently part of a local heat stroke prevention plan.
8. Local volunteer networks play a major role in dissemination of information and active outreach to vulnerable subgroups, particularly the elderly.
9. Preparedness of the social and healthcare systems are commonly not an explicit component of the plan.
10. Long term urban planning, though prevalent in most local government programs, is usually decoupled from heat risk protection initiatives (and usually ascribed to climate change mitigation strategies).
11. Reporting of heat-related outcomes and of selected indicators pertaining to the plan are common, but not a formal monitoring and evaluation either in terms of outcome or of process.
12. The personnel in charge of these programs do not usually have any formal and/or continuous interaction with prefectural or national authorities regarding this type of preventive activities.

Analyzed as systems, these heat-health prevention plans share attributes that are very relevant for public health management at the local level: (1) Easy and cheap to operate and maintain (2) Not labor intensive and (3) Strongly tied to local needs and resource availability. These attributes support the feasibility of local governments’ involvement in public health prevention against heat waves. 

A note on the intent and limitations of this exercise is important at this point. Our objective was to showcase selected examples of local public health activities for the prevention of heat waves in the specific context of Japan. Therefore, this review is not as rigorous or representative as a comprehensive assessment. Several heat-health action plans may exist in Japan that have not published information online. To our knowledge, there is not a publicly available registry of such systems in Japan. We tried to capture some of those plans based on the respondents of a questionnaire sent periodically by the Ministry of Health, Labor and Welfare (MOHLW) to several municipalities. That questionnaire attempts to collect information regarding local healthcare countermeasures to avoid heat disorders in the elderly and disabled. Results for the 2011 questionnaire responses are available online at the MOHLW website (http://www.mhlw.go.jp/topics/2011/06/dl/tp0629-1-1.pdf last accessed on 2 October 2011). Other researchers might want to conduct a comprehensive search based on this information. We simply did not have the time and resources needed to investigate these systems. 

Moreover, the analyzed plans have various target populations and scopes, so not all categories proposed by WHO apply uniformly to all selected case studies. Inevitably, some aspects or details about the plans may have changed from the time of the research to the publication stage. Details may have been lost in translation and/or slightly misinterpreted. However, regardless of such limitations, we feel that this review is relevant for two reasons: First, to raise attention on the opportunities for local governments to become effectively involved in public health adaptation to climate change impacts. And second, to highlight the work and initiatives of Japanese municipalities on heat waves public health prevention in order to promote a horizontal transfer of knowledge and ideas. 

Beyond their specific characteristics, the analysis of these five local heat stroke disorder prevention plans provides some key lessons for the involvement of municipalities in their own heat wave prevention. These lessons can also be applied in broader efforts towards their public health adaptation to climate change impacts:

1. Inter-sectoral coordination: this paper confirms previous observations [31] that health departments are not necessarily the only suitable lead agencies for heat disorder prevention at the local level, though health stakeholders should always play a major role in heat preventive efforts. Effective heat-health prevention and management requires strong inter sectoral coordination. Such coordination is most useful when combined with effective communication between different municipalities, as well as with higher levels of government and institutions.
2. Use of existing social systems and infrastructure: Social capital reservoirs such as volunteer organizations provide great opportunities for effective outreach to vulnerable populations without a large associated economic burden for local governments, a noteworthy matter, since lack of funding and personnel are common barriers for implementation of heat prevention activities [13].
3. Effective outreach: locally run heat-health prevention can effectively target preventive strategies to vulnerable populations and involving relevant stakeholders. For example, while email through PC may be useful for health care practitioners and institutions, individuals most at risk might not be frequent users; however, they might be reached through cell phone (more so in countries with high usage among people over 65, like Japan). Further, passive outreach (e.g., through leaflets) has proved ineffective for the elderly, the homeless and the socially isolated [32]. A strong existing community support system, the adequate regulatory framework and the widespread use of Information and Communications Technologies allows for an agile monitoring of at-risk individuals in Japan while safeguarding privacy and confidentiality. It also ensures that advice on how to keep cool is effectively translated to vulnerable groups.
4. Interaction with stakeholders: municipalities can assess their preparedness status through a strong and frequent communication with local health and social care providers. For instance, they can exchange information on the specific procedures hospitals, clinics, retirement and nursing homes adopt before and during the summer period and during heat-waves. When care providers are developing measures for improved cooling, the local environment or sustainability department may provide advice on ways of reducing the overall carbon footprint of the facility or institution.
5. Climate-friendly urban planning as a prevention strategy: while often programmatically unrelated to heat-health prevention, the efforts of local and prefectural governments in Japan to tackle climate change are very significant. The municipalities cited in this paper are no exception, and sub-national governments often promote or support the greening or urban spaces, increase in public water bodies and fountains, reduction of human-produced heat, the retrofitting of surfaces to increase albedo and sustainability-oriented lifestyle changes. While not all local governments may have the resources and incentives to undertake these activities, there are several options available to municipalities to manage their urban landscape in a way that reduces their inhabitants’ heat exposure in the short, medium and long term [33,34,35].

All these lessons support the idea of municipal involvement in this type of preventive action. Moreover, it is clear that national-level prevention is insufficient; even in a highly advanced society and strong welfare state like Japan, health effects from heat are increasing markedly. High urbanization rates and density, an aging population and climate change multiply the risks of heat waves. Short of radically changing the whole built environment of Japanese cities, further public health-based prevention will be necessary to avert some of these risks. Local governments can play a crucial role in the operationalization of public health protection measures for heat waves. 

For all the advantages of local involvement in heat disorder prevention, there is ample room for improvement in the way local governments plan for and respond to heat extremes [31,36]. Consistently, most local governments will probably need assistance for their public health adaptation planning, as suggested by a recent survey in the U.S. [37]. Our observations in Japan support those notions. Some of the dimensions in which national authorities could provide assistance to local governments are:

A. Scarcity of locally-relevant evidence: Despite the convenience of adopting ready-made national standards, heat risk thresholds should ideally be based on the analysis of local records on the relationship between temperature and mortality or disease. It is unclear whether general thresholds associated with increased mortality at the national level would prove equally effective in preventing heat deaths in diverse local settings. As an illustrative example, the complexity and diversity of risk factors has recently prompted several Japanese experts to propose a modification of the 1994 Japan Sports Association guidelines [38] by lowering WBGT limits. 

B. Lack of expertise: Whereas real time automated monitoring provides locally relevant indexes of risk, they cannot substitute for forecasts and expert judgment; the fast onset and high mortality associated with heat disorders requires triggering preventive actions as early as 1 or 2 days before the projected peak in risk [39]. Some activities included under local plans in Japan have no evidence supporting their preventive value. Such is the case for the “green curtain” projects, public misting places and fan distribution. Further, only a subset (the elderly) of all groups vulnerable to heat-related morbidity and mortality were explicitly addressed in most heat disorder prevention plans, leaving out the homeless, mentally ill, handicapped, the socially isolated and other subpopulations known to suffer during heat waves. In addition, local heat stroke prevention rarely includes the synergistic effects of air pollution, which has been observed to further increase mortality during heat waves [40].

C. Unsuitability for some dimensions of prevention: While local governments are in a good position to implement several heat-health preventive strategies, other institutional levels are better suited for many of the necessary tasks. Such is the case of epidemiologic surveillance, typically a resource-intensive and time-consuming task, better managed at the prefectural, regional, state or national level. 

## 4. Conclusions

Local governments should play a stronger role in public health adaptation to climate change impacts, and in particular in the prevention of health effects associated with heat waves in vulnerable areas Moreover, local governments and their public health departments are in a key position to shape and promote their own adaptation to climate change impacts. They can educate their communities and connect them to local, regional and national resources; organize local collective action and channel know-how and external support to different groups.

Additionally, some inherent characteristics give local governments advantages for a more effective public health protection from heat waves and other health risks increased by climate change. They can use their local social capital to boost the effectiveness of prevention, adapt existing systems to their own circumstances, communicate frequently with local stakeholders and use tools such as urban planning to protect communities against climate impacts. 

However, to play a significant role in public health adaptation, municipalities require support from national and regional governments in formats that are useful and tailored to their needs. In addition, different levels of governance need to coordinate their climate change policies, including adaptation actions, in order to maximize their effectiveness. 

More and clearer policy dialogue is needed between the different institutional levels and stakeholders involved in climate change adaptation for public health, both at the national and sub-national levels. The characteristics and functionality of existing heat disorder prevention efforts by municipalities can inform some dimensions of this dialogue. Further research is necessary to assess the status, effectiveness, weaknesses and opportunities of local preparedness and response to heat waves. 

## Annex 1. Structured List of Questions Asked to Local Government Officers Coordinating Heat-Health Action Plans

Note: because the questions were asked only to cities that run heat stroke prevention systems, there were no preliminary screening questions.

1. Leading Body

1.1. Is there one department that coordinates your city heat/health action plan? If so, what department?

1.2. Are there any other local government departments involved in the operation of the plan?

2. Alert System

2.1. What type of meteorological information and/or forecasts do you use to assess heat disorder risk? Where does that information come from and how often?

2.2. Who assesses the risk in your heat-health action plan? What criteria are used to assess the risk of heat disorders? Are risks categorized in different levels (e.g., no risk, moderate, high, *etc*.)? What is the meaning of each of these categories?

2.3. Is there a threshold temperature or index value to issue a risk warning? If so, how was that threshold determined? If there are any guidelines or models you followed, please mention them.

2.4. Who is in charge of monitoring this alert system? If there is an increased risk of heat disorders, is a warning system triggered? Is the system automatic or manual? If manual, who triggers the system and how? If automatic, how is it set up?

3. Heat-Related Health Information Plan

3.1. Does the city government promote awareness of the health risks of heat? How?

3.2. Is the city government disseminating advice on how to avoid health effects from heat? Is behavioural advice included in the distributed health information?

3.3. When a heat risk warning is triggered, who does the city government notify? How does the notification system work? Which actions are taken routinely? Which actions are taken only in “Alert days”?

4. Reduction in Indoor Heat Exposure

4.1. Does the plan include short-term and medium-term strategies to reduce heat exposure for residents? (This includes actions at both the individual and local community levels.) (e.g., air-conditioned public spaces, advice on how to keep houses or apartments cool, simple advice for residence cooling, *etc*.)

5. Care for Vulnerable People

5.1. Does the plan include specific provisions or actions to protect vulnerable groups, such as young children, the elderly or the chronically ill, from heat? If so, what are those provisions or actions? What outreach strategies are used, if any?

5.2. Does the city government communicate or interact with the management of residences for the elderly, long-term care facilities and shelters regarding heat risks? Please describe such interaction and/or communication. Are these included in the heat-health prevention plan?

6. Preparedness of the Health and Social Care System

6.1. Does the plan include measures to increase preparedness for heat risks of local healthcare and or social care providers? (Examples include staff training, contingency plans, distribution of guidelines for healthcare and social care providers, *etc*.)

7. Long-Term Urban Planning

7.1. Besides the public health prevention efforts against heat, is the city government undertaking any actions in urban planning, urban design or infrastructure that could prevent or reduce hazardous heat exposures? (Examples include green roofing, better building insulation, tree planting, building wind corridors within the city, increasing green spaces within the city, *etc*.)

8. Surveillance and Evaluation

8.1. Does your city government collect information on heat-related disorders? (e.g., Hospital admissions, ambulance calls, *etc*.) Is that collection of information included in the plan?

8.2. If health data are collected, is this information reported to other entities?

8.3. Are there (to your knowledge) any institutions or facilities outside the city government that collect such health data? Please list the institutions/facilities as well as their roles. Is that information shared with the city government?

8.4. Is the plan evaluated periodically? Based on what indicators and/or information?

9. Interaction with Other Levels of Government

9.1. Does the city government have any interaction with prefectural and/or national authorities regarding the prevention of health effects from heat? If so, is that communication periodical? Please describe any interaction regarding this topic with said authorities.
